# Editorial: The development of biomarkers in psychiatry

**DOI:** 10.3389/fpsyt.2022.1075993

**Published:** 2022-12-01

**Authors:** Takashi Nakano, Masahiro Takamura, Takahiro A. Kato, Shin-ichi Kano

**Affiliations:** ^1^Department of Computational Biology, School of Medicine, Fujita Health University, Toyoake, Japan; ^2^Division of Computational Science, International Center for Brain Science, Fujita Health University, Toyoake, Japan; ^3^Department of Neurology, Faculty of Medicine, Shimane University, Izumo, Japan; ^4^Department of Neuropsychiatry, Graduate School of Medical Sciences, Kyushu University, Fukuoka, Japan; ^5^Department of Psychiatry and Behavioral Neurobiology, Heersink School of Medicine, University of Alabama at Birmingham, Birmingham, AL, United States

**Keywords:** psychiatry, major depressive disorder, attention-deficit hyperactivity disorder (ADHD), post-traumatic stress disorder (PTSD), biomarkers

Psychiatric disorders are diagnosed primarily through interviews and observations. This makes it difficult to correctly diagnose patients and select an appropriate treatment at the first visit. Thus, there is an urgent need for more objective indicators based on biological evidence. Biomarkers are objective indicators of disease presence, disease status changes, and treatment effectiveness. To date, a wide range of biomarkers, such as molecules, proteins, and physiological activities, including brain activity, have been developed. This Research Topic includes nine recent studies on the development of biomarkers for psychiatric disorders and one opinion. The proposed potential biomarkers include metabolomic profiles of blood or urine, electroencephalography (EEG) and functional magnetic resonance imaging (fMRI) measurements, volatile organic compound (VOC) profiles in exhaled breath, vocal acoustic features, and visual evoked potentials. This Research Topic multiple disorders, including major depressive disorder (MDD), post-traumatic stress disorder (PTSD), and attention-deficit hyperactivity disorder (ADHD).

Among the studies targeting MDD, Xie et al. measured resting-state brain activity using fMRI. Their results suggested disrupted causal connectivity among brain networks, including the default mode network, in drug-naive first-episode MDD patients. Sun et al. also used resting-state fMRI for patients with MDD, focusing on treatment-resistant and non-treatment-resistant depression. They found differences in some indicators of brain activity, low-frequency fluctuations, and regional homogeneity between the groups. Du et al. used visual evoked potentials, electrical signals generated at the visual cortex by a visual stimulus. Their findings suggest changes in the excitation-inhibition balance of the visual cortex in patients with MDD.

Indicators other than brain activity have also been suggested as potential biomarkers. Lueno et al. measured VOC concentrations in the exhaled breath of patients with MDD and demonstrated the possibility of using VOCs as promising biomarkers. Zhao et al. reported that vocal acoustic features could be potential biomarkers of MDD. They found altered acoustic expressions of emotion in MDD patients compared to healthy controls, suggesting a relationship between acoustic characteristics and the severity of depressive symptoms. Höller et al. investigated seasonal affective disorders using non-clinical samples. They used questionnaires and brain activity measured using EEG to predict mood decline. Höller et al. also showed that seasonality interacts with age and EEG power within the alpha frequency range.

Regarding psychiatric disorders besides mood disorders, Zhu et al. investigated the relationship between ferroptosis, iron-dependent regulated cell death, and PTSD. They applied machine-learning algorithms to blood transcriptome data and successfully predicted PTSD with ferroptosis-related genes. Tian et al. performed urinary metabolomic profiling to diagnose ADHD in children and adolescents. Levels of urine metabolites differed between patients with ADHD and healthy controls. They applied machine learning to predict ADHD using urinary metabolites and succeeded in constructing a model with good predictive ability. Myint and Halaris discussed the kynurenine pathway as a potential biomarker of psychiatric disorders. They also introduced esketamine and its possible therapeutic roles since esketamine is the only currently available medication that is directly linked to the role of the kynurenine pathway in psychiatric disorders.

Many of these studies investigated differences in biological characteristics between patients and healthy subjects or treatment-resistant and non-resistant groups; furthermore, some studies used machine learning to predict the prognosis or diagnosis. Combining biological evidence and machine learning makes it possible to predict the disorder or treatment response of a person (1). The development of machine learning and artificial intelligence has enabled us to increase the predictive value of biomarkers (2). Moreover, biomarkers can be used to predict treatment effects, which leads to tailor-made medicine according to individual characteristics (3), such as neurofeedback (4). New molecular biomarkers, such as cell-free nucleic acids and extracellular vesicles, are actively investigated (5, 6). Expanding research on the development of biomarkers will contribute to a better diagnosis and a deeper understanding of psychiatric disorders.

## Author contributions

TN wrote the manuscript. All authors contributed to the article and approved the submitted version.

## Conflict of interest

The authors declare that the research was conducted in the absence of any commercial or financial relationships that could be construed as a potential conflict of interest.

## Publisher's note

All claims expressed in this article are solely those of the authors and do not necessarily represent those of their affiliated organizations, or those of the publisher, the editors and the reviewers. Any product that may be evaluated in this article, or claim that may be made by its manufacturer, is not guaranteed or endorsed by the publisher.
